# Rethinking treatment approaches for FIGO stage IVB cervical cancer: personalized strategies and emerging therapies

**DOI:** 10.3389/fimmu.2025.1567296

**Published:** 2025-04-08

**Authors:** Jing Zeng, Rutie Yin

**Affiliations:** ^1^ Department of Obstetrics and Gynecology, West China Second University Hospital, Sichuan University, Chengdu, China; ^2^ Key Laboratory of Birth Defects and Related Diseases of Women and Children, West China Second University Hospital, Sichuan University, Chengdu, Sichuan, China; ^3^ Department of Gynecology and Obstetrics, West China Second University Hospital, Sichuan University, Chengdu, China

**Keywords:** cervical cancer, stage IVB, immunotherapy, localized treatment, personalized strategies

## Introduction

FIGO stage IVB cervical cancer (IVB-CC), characterized by tumor spread beyond the true pelvis with distant metastases, represents one of the most challenging subsets of cervical cancer to treat (1, 2). Despite advances in systemic and localized therapies over the past decade, the prognosis for IVB-CC remains poor, with a 5-year survival rate of only 14.7% (3). While trials such as KEYNOTE-826 and BEATcc have shown the potential of immunotherapy as a first-line option for persistent, recurrent, and metastatic cervical cancer, they reveal significant gaps when applied to IVB-CC patients specifically. This commentary examines these gaps, emphasizes the importance of tailored therapeutic strategies, and proposes directions for future research.

## Challenges of first-line immunotherapy

Immunotherapy has become a transformative treatment for various cancers, including cervical cancer. However, its benefits for IVB-CC patients are far from clear. The KEYNOTE-826 trial evaluated pembrolizumab in combination with chemotherapy (with or without bevacizumab) in patients with persistent, recurrent, and metastatic cervical cancer (4, 5). The median progression-free survival (PFS) for the combination therapy group was 10.4 months, compared to 8.2 months for the control group (hazard ratio [HR] 0.65 [95% CI: 0.53–0.79]). The median overall survival (OS) was 26.4 months versus 16.8 months (HR 0.63 [95% CI: 0.52–0.77]), respectively. While the trial demonstrated significant benefits for the overall study population, subgroup analyses raised questions regarding the applicability of its findings to specific patient subsets. Patients with programmed cell death protein ligand 1 (PD-L1) expression <1% showed no statistically significant differences in PFS (HR 0.94 [95% CI: 0.52–1.70]) or OS (HR 0.87 [95% CI: 0.50–1.52]). Similarly, patients with metastatic disease at diagnosis derived no significant PFS (HR 0.92 [95% CI: 0.64–1.30]) or OS (HR 0.85 [95% CI: 0.60–1.21]) benefit from the combination therapy. Based on these findings, the National Comprehensive Cancer Network (NCCN) guidelines recommend pembrolizumab for advanced, recurrent, and metastatic patients with PD-L1 expression ≥1. However, they fail to address the lack of benefit observed in patients with metastatic disease at initial diagnosis, raising concerns about the blanket applicability of this recommendation to IVB-CC patients.

Similarly, the BEATcc trial examined the combination of atezolizumab, cisplatin/paclitaxel, and bevacizumab in patients with IVB, persistent, and recurrent cervical cancer (6). The results showed significant improvements in PFS and OS for the combination therapy group, with a median PFS of 13.7 months compared to 10.4 months for the control group (HR 0.62 [95% CI: 0.49–0.78]) and an OS of 32.1 months compared to 22.8 months for the control group (HR 0.68 [95% CI: 0.52–0.88]). However, subgroup analyses revealed that IVB-CC patients with distant metastases showed no statistically significant improvement in PFS (HR 0.71 [95% CI: 0.43–1.16]) or OS (HR 0.85 [95% CI: 0.49–1.49]). Similarly, patients without prior chemoradiotherapy (CRT) failed to show significant benefits in PFS (HR 0.77 [95% CI: 0.52–1.12]) or OS (HR 0.86 [95% CI: 0.55–1.34]). These findings highlight the substantial variability in treatment responses among different subpopulations of cervical cancer, underscoring the need for more tailored approaches in IVB-CC management.

A recently published meta-analysis evaluated eight Phase III trials, including BEATcc and KEYNOTE-826, to address the challenges of interpreting evidence for IVB-CC specifically (7). Of the eight trials, only three included IVB-CC as a defined subgroup (KEYNOTE-826, BEATcc, and GOG240). Despite the introduction of novel therapies, the pooled HR for OS in IVB-CC patients remains 0.85 (95% CI: 0.64–1.14), indicating no significant survival improvement compared to persistent and recurrent cervical cancer (HR 0.64 [95% CI: 0.55–0.75]), highlighting the unique challenges this subgroup faces.

These findings challenge the current practice of extending broad immunotherapy recommendations to IVB-CC patients as first-line treatment without sufficient evidence. IVB-CC differs fundamentally from persistent or recurrent cervical cancer in that patients often present with no prior history of surgical, radiation, or systemic therapy. This crucial distinction highlights the inappropriateness of treating IVB-CC as equivalent to other advanced-stage disease categories when formulating treatment guidelines.

## Role of localized treatment in IVB-CC

IVB-CC encompasses a spectrum of disease presentations, from single distant organ metastases or oligometastatic disease to widespread multi-organ involvement. A retrospective cohort study of 1,772 women with stage IVB cervical cancer revealed significant survival differences based on metastatic extent, with bone metastasis alone (median OS: 11 months) associated with better outcomes than multiple organ involvement (median OS: 3 months; absolute difference 8 months, P<0.001) (8). Similarly, another study highlighted the prognostic impact of metastasis type, demonstrating that patients with hematogenous metastases had worse outcomes, with a 3-year OS rates of 20%, compared to lymphatic metastases, which showed the rates of 57.2% (P=0.017) (9). Therefore, even within the broad category of IVB-CC, differences in the number and type of metastases highlight the need for distinct treatment strategies tailored to each patient’s metastatic profile.

Emerging evidence suggests that certain subgroups of IVB-CC patients, particularly those with oligometastatic disease, may derive significant survival benefits from localized interventions such as radiotherapy. A retrospective study involving 60 patients with synchronous oligometastatic IVB-CC demonstrated that definitive irradiation targeting both primary and metastatic sites, delivered through Volumetric Modulated Arc Therapy (VMAT) or intensity-modulated radiation therapy (IMRT), followed by intracavitary/interstitial brachytherapy, resulted in a 5-year OS rate of 51.4% and a 5-year PFS rate of 25.9%. Patients with a single metastatic site showed markedly better outcomes, with a 3-year OS rate of 60.4% compared to 20.6% for those with ≥2 metastatic sites (p = 0.003) (9). Furthermore, a multi-institutional retrospective study revealed that combining whole pelvic radiation (WPR) with chemotherapy significantly extended median OS (41.6 months vs. 17.6 months, p < 0.01) in stage IVB cervical cancer patients, without an increase in complication rates (10). Similarly, a systematic review of eight retrospective studies involving 2,424 patients reinforced the survival advantage of definitive pelvic radiotherapy over systemic chemotherapy (with or without palliative radiotherapy) (11). Patients receiving radiotherapy had significantly improved median OS, ranging from 14 to 63.7 months, compared to 10 to 19 months in the chemotherapy group, favoring the groups that received definitive pelvic radiotherapy. These findings underscore the potential of radiotherapy to improve outcomes in IVB-CC, though prospective trials are needed to validate its role in standard practice.

Currently, the majority of scholars consider surgery no longer an option for IVB-CC, and systemic treatment is typically the first-line approach. However, recent population-based real-world data have provided some new insights. A population-based retrospective study of 434 patients demonstrated that primary tumor resection (PTR) reduced the cancer related and overall mortality rates by 31% and 30%, respectively, after propensity score matching and adjusting confounders (12). Another population-based study found that PTR combined with CRT significantly improved overall survival in stage IVB cervical cancer patients, particularly for those without visceral metastasis (13). These findings underscore the potential benefit of surgery in improving survival, particularly for patients with limited metastatic disease, and highlight the need for stratified treatment strategies in managing IVB-CC. However, the evidence level of these studies is relatively low and cannot yet guide clinical practice. Prospective randomized controlled trials (RCTs) are still needed to validate these findings.

## Conclusion and proposes directions for future research

The treatment of IVB-CC still faces many unresolved issues. One major question is whether systemic chemotherapy or radiotherapy should be prioritized, or if a combination of both offers better outcomes. Another key concern is the timing of immunotherapy—whether it should be used as a first-line treatment to benefit patients early, or delayed until disease progression or recurrence for greater efficacy. Additionally, the role of localized treatments, such as radiotherapy or surgery, in oligometastatic or selected patient populations is often overlooked. However, there is currently no well-designed prospective study that validates the role of radiotherapy or localized surgery in IVB-CC. Furthermore, unresolved issues remain regarding the optimal dose of radiotherapy, the role of brathytherapy and whether it should be combined with targeted therapy or immunotherapy.

At the same time, the heterogeneity of IVB-CC calls for a shift from a one-size-fits-all approach to personalized treatment strategies. Patient stratification based on metastatic patterns, disease burden, and specific biomarkers is essential. Treatment decisions should be guided by a combination of clinical features, such as the number and location of metastases, and molecular biomarkers. For example, while PD-L1 expression remains a significant marker for immunotherapy selection, it is insufficient on its own to predict responses in IVB-CC due to the observed variability in treatment outcomes. Other promising biomarkers to consider include microsatellite instability (MSI), tumor mutational burden (TMB), and specific genetic mutations, such as those related to HPV-driven carcinogenesis or DNA repair pathways. Future research should prioritize the identification of additional biomarkers, leveraging genomic and transcriptomic data to refine treatment selection. This will allow for a more tailored approach, offering better chances of response to specific therapies.
